# Ultrasound Simulation for Training Trainees when the Luxury Becomes Essential: Opinion and Evidence Obtained during the Latvian Research Council Project Implementation

**DOI:** 10.15388/Amed.2022.29.2.16

**Published:** 2022-06-29

**Authors:** Natālija Vedmedovska, Anda Ķīvīte-Urtāne, Ija Lisovaja, Laura Rācene, Līva Ķīse, Beāte Sārta, Agnija Vaska, Zane Rostoka, Violeta Bule, Ieva Pitkēviča, Dace Rezeberga

**Affiliations:** Riga Stradiņš University, Department of Obstetrics and Gynecology, Rīga, Latvia; Riga Maternity Hospital, Rīga, Latvia; Riga Stradiņš University, Institute of Public Health, Rīga, Latvia; Riga Stradiņš University, Department of Public Health and Epidemiology, Rīga, Latvia; Riga Stradiņš University, Department of Obstetrics and Gynecology, Rīga, Latvia; Riga Maternity Hospital, Rīga, Latvia; Riga Stradiņš University, Department of Obstetrics and Gynecology, Rīga, Latvia; Riga Maternity Hospital, Rīga, Latvia; Riga Stradiņš University, Department of Obstetrics and Gynecology, Rīga, Latvia; Riga Maternity Hospital, Rīga, Latvia; Riga Stradiņš University, Department of Obstetrics and Gynecology, Rīga, Latvia; Riga Maternity Hospital, Rīga, Latvia; Riga Stradiņš University, Department of Obstetrics and Gynecology, Rīga, Latvia; Riga Maternity Hospital, Rīga, Latvia; Riga Stradiņš University, Department of Obstetrics and Gynecology, Rīga, Latvia; Riga Maternity Hospital, Rīga, Latvia; Riga Stradiņš University, Department of Obstetrics and Gynecology, Rīga, Latvia; Riga Maternity Hospital, Rīga, Latvia; Riga Stradiņš University, Department of Obstetrics and Gynecology, Rīga, Latvia; Riga Maternity Hospital, Rīga, Latvia; Riga Stradiņš University, Department of Obstetrics and Gynecology, Rīga, Latvia; Riga Maternity Hospital, Rīga, Latvia

**Keywords:** training, trainees, simulation, confidence, practical skills

## Abstract

**Background:**

Simulation as a proxy tool for conditional clinical training became a powerful technique for introducing trainees to the ultrasound imaging world, allowing them to become a trained sonographer taking into consideration different rates of progress in completing a specific task against the time and ensuring the long-lasting maintenance of the obtained practical skills. Adding a costly, but effective high-fidelity simulator to the residency program justified the expense, demonstrating efficiency of training for improving the clinical performance and confidence of trainees.

**Materials and methods:**

A pilot study in Riga Maternity Hospital within the framework of the study “Role of metabolome, biomarkers and ultrasound parameters in successful labor induction” (Fundamental and Applied Research Programme lzp-2021/1-0300) was performed between March 1st 2022 and 31st April 2022. A virtual-reality simulator (ScanTrainer, Medaphor^TM^, Cardiff, UK) was used with the teaching module for assessment of the uterine cervix. Five trainees in obstetrics and two young specialists included in the study. None of them had Fetal Medicine Foundation certificate of competence in the assessment of the uterine cervical lenght before. The time used on the simulator, the number of simulations and a mean confidence in cervical length assessment before and after simulation were recorded.

**Results:**

The study on assesment of uterine cervical lenght demonstrated statistically significant increase in confidence (p=0.008) and statistically significant decrease in time needed to complete correctly the same tasks for the trainees (p=0.008) that shows a positive learning curve over the time of training on ScanTrainer, Medaphor.

**Conclusions:**

The simple task allows to become a certified specialist in uterine cervical assessment in the short period of time. That support the productiveness of the simulation-based education. The training program should be updated taking into consideration simulation curriculum.

## Santrauka.

**Kontekstas:**

Simuliacija yra atstovaujamoji sąlyginio klinikinio mokymo strategija, kuri jau tapo veiksmingu metodu įtraukti besimokančiuosius į ultragarso atvaizdų pasaulį. Tai jiems leidžia tapti mokytais sonografijos specialistais, nes atsižvelgiama į skirtingą pažangos spartą ir konkrečios veiklos atlikimo laiką. Kartu užtikrinama, kad įgyti praktiniai įgūdžiai bus ilgalaikiai. Aukštos raiškos ir kokybės simuliatoriaus naudojimas rezidentų programoje yra brangi, tačiau veiksminga priemonė. Išlaidos pasiteisino, ir tyrimas atskleidė tokio mokymo veiksmingumą, nes pagerėjo ne tik besimokančiųjų klinikinės veiklos rezultatai, bet padidėjo jų pasitikėjimas savimi.

**Medžiagos ir metodai:**

Bandomasis tyrimas Rygos motinystės ligoninėje programos „Metabolomo, biožymenų ir ultragarso parametrų vaidmuo sėkmingai susipažįstant su darbo procesu“ kontekste (Fundamentaliųjų ir taikomųjų tyrimų programa lzp-2021/1-0300) buvo vykdytas nuo 2022 m. kovo 1 d. iki balandžio 30 dienos. Mokymo modulyje naudotas virtualios realybės simuliatorius (*ScanTrainer, MedaphorTM*, Kardifas, Jungtinė Karalystė), juo buvo vertinama gimdos kaklelio būklė. Tyrime dalyvavo penki akušerijos studentai ir du jauni specialistai. Nė vienas iš jų tyrimui prasidedant dar neturėjo Vaisiaus medicinos fondo kompetencijos sertifikato, leidžiančio vertinti gimdos kaklelio ilgį. Buvo registruojamas laikas, praleistas dirbant su simuliatoriumi, simuliacijų skaičius ir vidutinis pasitikėjimo lygis gimdos kaklelio vertinimu prieš ir po simuliacijos.

**Rezultatai:**

Gimdos kaklelio ilgio vertinimas atskleidė statistiškai svarbų pasitikėjimo savimi padidėjimą (p = 0,008) bei statistiškai svarbų sumažėjimą laiko, būtino besimokantiesiems, kad jie taisyklingai atliktų tas pačias užduotis (p = 0,008). Tai atskleidžia, kad mokymosi su *ScanTrainer, Medaphor* laikotarpis užtikrino pozityvius rezultatų pokyčius.

**Išvados:**

Ši paprasta užduotis leidžia tapti sertifikuotu gimdos kaklelio vertinimo specialistu per trumpą laiką. Buvo atskleistas simuliacija grindžiamo mokymosi produktyvumas. Mokymosi programa turėtų būti atnaujinta atsižvelgiant į simuliacijų mokymosi planą.

## Introduction

There is no doubt that ultrasound examination has a crucial role in modern patient care in obstetrics and gynecology, including emergency, standard and follow-up evaluation [1]. The method is approved to be safe and sensitive, but as any other diagnostic imaging modality it is highly dependent on skills and experience of its operator [2]. Consequently, inadequate expertise causes missed and wrong diagnosis, erroneous healthcare procedures and needless anxiety of patients [3].

Ultrasound skills are time and effort consuming and associated with a long learning curve to achieve independent practice. For example, to obtain competent skills for the first trimester screening in detection of major malformations, it is necessary to perform 15 examinations per week or 700 examinations per year during 3–4 years [4]. More recent studies demonstrated that following standard protocols, suggested by the International Society of Ultrasound in Obstetrics and Gynecology (ISUOG), examiners with different skill levels have the ability to reach 95% successful visualization of fetal structures at the first trimester after training on 50 cases [5]. Similarly, researchers tried to evaluate the learning curves in assessing pelvic disorders. Burgetova et al. confirmed that evaluation of approximately 100–150 cases of pelvic endometriosis is required to achieve a plateau of knowledge in pelvic anatomy or assessment of 35 cases of deep endometriosis (DE) to achieve a sufficient level of competency in predicting pouch of Douglas (POD) obliteration and bowel DE [6]. Leonardy et al. [7] came to the similar conclusion affirming that it is feasible for some gynecologic fellows, with varying or limited experience in gynecologic ultrasound (US), to detect obliteration of POD and DE of the bowel in a program of 50 examinations. Therefore effective, sequential, systematic and continuous training in ultrasound is essential to supply skillful examinations.

### Incorporating of ultrasound simulation at the educational system

Historically ultrasound methods have been taught by hands-on practical manner training on patients or volunteers, but ethical issues of multiple unnecessary vaginal scans in gynecologic settings and As Low As Reasonably Achievable (ALARA) principle in obstetrics make these clinical training approaches less relevant. Moreover, the learning ability of the trainees, their pace of assimilating and incorporating knowledge and practical competence are very variable in such a setting [8,9].

The concerning rise of professional malpractice cases and patient’s reluctance to be interrogated by unskilled trainees brought on the stage the auxiliary technique for consecutive training in a realistic, but artificial environment, that allows both to develop competency in ultrasound, and assess skills by receiving feedback on the level of expertise [10].

This new educational system incorporates ultrasound simulators, putting together phantoms, probe replicas and any devices with an operating system that leads to a realistic simulation of clinical scenarios, generating required haptic experience, probe orientation skills as well as correct image acquisition and optimization without discomfort or misuse of patients [11,12]. Moreover, the repetitions of simulation are timeless and without limitation. Not only sophisticated costly ultrasound simulation systems, but also “low cost” browser-based applications were created and available for training out of the clinical settings and are proven to be superior to conventional teaching [13]. Despite a variety of guidelines and recommendations of European Federation of Societies for Ultrasound in Medicine and Biology (EFSUMB) concerning ultrasound, extensive ultrasound core curriculum and training programs are still diverse and not uniform in European countries [1]. According to the EFSUMB recommendations the basic course should be conducted as a multidisciplinary lesson module followed by at least 9 months advanced (under the supervision of a certified ultrasound operator) and final ones in specific obstetrics and gynecology (ObGyn) areas.

### The first attempt of incorporating of ultrasound simulation in Latvian residency program and the primary results

The existing national training program of ultrasound imaging for residents in gynecology and obstetrics in Latvia is already integrated as a core subject, to which up to 20 weeks is devoted, combining basic ultrasound activity in outpatient settings and advanced in referral hospitals. For certification a trainee should have 60 gynecological and 150 obstetric scans and a logbook with 43 ultrasound images, based on Project for achieving Consensus in Training by the European Board and College of Obstetrics and Gynecology [14].

Unfortunately, the use of newer ultrasound technological developments for training purposes, including simulation-based practical training has not yet been applied to date in Riga Stradiņš University. Consequently, most hands-on supervision of residents in departments are nowadays performed in a rather unstructured way [15]. In such a setting, a clinical supervisor would typically oversee the trainee’s performance or repeat the scan in cases of request. As the motivation to teach differs vastly between supervisors, this can explain the large discrepancies between ObGyn trainees’ confidence and levels of accomplishment in performing ultrasound examinations at the end of the residency, and at the same time mandates for an update of ultrasound training in our country.

Relating to the certified ultrasound examination there are specific national considerations, too. In Latvia gynecological and obstetrical ultrasound examinations are nowadays allowed to be performed by physicians only. Compulsory prerequisites for professional ultrasound qualifications in gynecology and obstetrics can be acquired by a combining all the following activities:

1) attending ultrasound courses, webinars or other web-based resources and interactive tools and collecting CME credits; 2) performing up to 1000 gynecological and obstetrical examinations (logbooks of completed examinations); 3) an image-based acceptance test must be carried out to achieve approval for those, who has an intention to perform the first trimester screening studies and 4) passing a final theoretical exam.

For reaccreditation, all physicians who were previously licensed for ultrasound performance in obstetrics and gynecology must document 1000 ultrasound examinations (logbooks of completed examinations), attend ultrasound courses, webinars or other web-based resources and interactive tools corresponding to the area of subspeciality and collect CME credits.

Unfortunately, as stated before [1], lectures, technical videos and various archives of clinical cases cannot replace the vital hands-on training, practicing a systematic approach in acquiring correct planes and image assessment, followed by the subsequent correct clinical decision-making. Therefore, the level of ultrasound specialists and quality of examinations are diverse within the country and seems to be worse in the rural regions, compared to that available in the central hospitals.

Recently we have performed a pilot study about an interactive ultrasound simulation on cervical length assessment. The uterine cervix is the lower part of the uterus and, recently, assessment of the cervix by ultrasound has become an essential part of diagnostic imaging in obstetrics.

## Materials and methods

The study originated in Riga Maternity Hospital within the framework of the study “Role of metabolome, biomarkers and ultrasound parameters in successful labour induction” (Fundamental and Applied Research Programme lzp-2021/1-0300) between March 1st 2022 and 31st April 2022. The virtual reality simulator ScanTrainer (Medaphor^TM^, Cardiff, UK) was used. The study group consisted of 7 participants. Five ot them were trainees in obstetrics and gynecology, who were undergoing specialty training, from whom one participant was of the 1st year of residency, three of the second and one of the 4th. Two young specialists with 1 and 2 years of experience and varying US experience were included as well. There was no special practical US training on uterine cervix assessment during residency. No fellow had formal training or experience in advanced transvaginal ultrasound (TVUS) for assessment of cervix and its length. The doctors who had finished their residency had 10 to 12 weeks of ObGyn ultrasound training in US unit, but none of them had Fetal Medicine Foundation (FMF) certificate of competence in cervical assessment before this study. The FMF has introduced different educational programmes for professionals and a series of certificates of competence in different area of fetal medicine, including cervical assessment [16].

The research questions were: 1) How does training by simulation and amount of time spent on it affect trainees’ level of confidence in assessment of cervix? 2) How fast, if at all, is it possible to get the Fetal Medicine Foundation certificate on cervical assessment after simulation-based training?

## Statistical analysis

Data analysis was performed using SPSS 26.0 software. The goodness of fit of the data to a normal distribution was checked with Kolmogorov–Smirnov test. Related-samples Wilcoxon Signet Rank test was used to assess the statistical significance of differences between pre- and post-intervention measurements. Results were considered as statistically significant if p<0.05.

After attendance of the FMF internet based theoretical course on cervical assessment [16] the trainees were provided with a 40-min introduction to the simulated environment and equipment [17], during which an examination and assessment of uterine cervix was demonstrated. The participants underwent training alone but on request the verbal feedback was provided by one of two simulation instructors (N.V. or I.L.). The participants were required to train on selected modules until they passed a predefined expert level of performance corresponding to the maximum total score 5. All virtual reality simulator training was dispersed in sessions of unlimited duration and took place in Riga Maternity Hospital Simulator center of Rīga Stradiņš University. The time used on the simulator, the number of simulations and a mean confidence in assessment of uterine cervix before and after simulation were recorded.

## Results

The first part of the results of the study addressed confidence in measurement of cervix length before and after the completion of simulation scans and the time (in min) needed for each trainee in order to complete the practical and test module (Table 1).

**Table 1.** Confidence in uterine cervix length measurement before and after the completion of simulation scans and time (in min) for each trainee needed to complete the practical and test module.

Trainee	Confidence before, max 5	Confidence after, max 5	p value	Practical module, min	Test module, min	p value
1	3	5	0.008	46	36	**0.008**
2	3	4		50	17	
3	3	5		40	11	
4	2	3		113	23	
5	3	5		46	25	
6	2	4		53	16	
7	3	4		70	17	

The median confidence (score 1 to 5) in uterine cervix length measurement was 3 before and 4 after simulation. No participant had a decrease or even the same level in confidence. Every participant had an increase in confidence of 1 to 2 points, and overall, there was a significant increase in confidence (p=0.008). Three out of 7 trainees rated maximal confidence (score of 5) after the simulation training, while one trainee with lowest confidence before start of simulation (score 2) increased confidence with 1 point only to a score of 3 after simulation.

The time trainees spent on theory module with demonstration video ranged from 8 to 58 min. The median time needed for the trainee to complete the practical module of 4 tasks was 50 min, while this was 17 min to finish the test module of 4 tasks. The test module included the same tasks as the practical module. Therefore, there was a significant decrease in time needed to complete the same tasks after training (p=0.008), indicating a positive learning curve after using ScanTrainer. The trainee with the lowest confidence scores before and after simulation training also needed more than double the time to perform the practical module (113 min vs. 51 min (mean), but finally passed the test at a similar time as all the others (23 min vs. 25 min (mean).

The number of simulations that were needed to complete one correct scan on uterine cervix assesment ranged from 1 to 15 simulation attempts. For the first task, the number of attempts ranged from 3 to 15 times and in the fourth task it ranged from 1 to 3 times. However no staistical difference was found in number of attempts between practical and test module despite the significant decrease in time.

Once trainees reached the expert level of performance on the virtual reality simulator, they applied for the FMF certificate [16] by submitting 2 images of unsupervised transvaginal scans of the cervix during pregnancy. Eligible patients for the clinical performance were emergency patients who were referred to obstetrical department of Riga Maternity hospital. The recordings were made while participants were on call. The time of licensing varied from 2 weeks to 2 months, depending on the training year and placement of training. All trainees succeeded to obtain the FMF certificate after their first attempt of submission and now are on the list of FMF licensed specialists on sonographic measurement of cervical length of the uterus. This tool is useful in the prediction of early preterm delivery, distinction between true and false labor in case of threatened preterm labor, and in the prediction of likelihood of cesarean section after induction of labor [16].

## Discussions

Our small pilot study clearly demonstrates the potential efficiency of a high-fidelity simulator, for improving the clinical performance and confidence of the participants in accomplishing sonographic techniques on real patients, regarding assesment of uterine cervix in pregnant woman. This corresponds with the findings of Byford et al. [9] study when even unexperienced trainees had an increase in confidence.

Moreover, the simulator not only helps to improve the management of patients, but also lowers the anxiety of trainees when engaging on the first transvaginal scans during their first years of residency. It is inevitable that this tool will become increasingly indispensable in future training of residents in ObGyn. Although its cost may seem unsurmountable, in long term it saves time of people management, which is less optimal and more costly. So, we believe that adoption of simulators in the residency program is an economically favorable investment in the long run and will offer great advantages for residents before entering on-call shifts in hospitals. Furthermore, the adoption of such programs offers the opportunity to standardize the training, as performing these tests are independent of the quality of training that is given by human experts, who may not always have the time, will and skills to teach such techniques properly. The same principle could be adapted in the radiological training program. Both studies of Ahmad et al. [18] and Rees et al. [19] found that ability of radiology residents to evaluate gynecological structures increased significantly after simulation.

To summarize, it is important to continuously update the residency program in obstetrics and gynecology and to build a comprehensive ultrasound core curriculum. For the latter, educational theoretical modules and simulation training syllabus knowledge must be carried through with feedback to the student at the end of every exercise. Using a simulator is a perfect alternative tool for evaluation of trainees and the results of trainees correspond to the evaluation on the patient model [20]. For example, during the first year of residency the basic course, that allows trainees to become familiar with knobology, probe orientation, image optimization (gain, zoom, setting of calipers), image or video storage and recordings, as well as probe disinfection and machine maintenance, would include an interdisciplinary course and a point-of-care ultrasound module. During the second year of residency, the first trimester normal anatomy and module of the standard second trimester biometry simulation could be accomplished. Le Lous et al. reported a significant reduction in the time to obtain the right planes and the time taken for trainees to complete CRL and fetal biometry scans with multiple tasks repetitions [21]. Therefore, this approach will not only facilitate the examination and necessary measurements, but also provide the capability to perform detailed anatomical evaluation and recognize the deviation from the normal features according to the perinatal medicine foundation (PMF) and the World Association of Perinatal Medicine (WAPM) guidelines [22] and will give the possibility to obtain FMF certificate of early screening for chromosomal abnormalities.

As already stated, adherence to examination protocols is associated with higher detection rates for fetal structural anomalies in all trimesters [23]. To complete the third- and fourth-years of residency, simulation scenarios of abnormal 1st and 2nd trimesters, as well as advanced gynecological cases must be undertaken, practicing decision making and tests ordering in evidence based and cost-effective manner. Taking into consideration the increasing importance of intrapartum ultrasound, the evaluation of posterior cervical and maternal subpubic arch angles, as well as the head–perineum distance, the angle of progression and other advantageous parameters of ultrasound pelvimetry [24] should be included into the curiculum of the last year of residency.

## Conclusions and future direction

Simulation as a proxy tool for conditional clinical training is becoming a powerful technique for introducing trainees to the ultrasound imaging world, allowing them to become a trained sonographer taking into consideration different rates of progress in completing a specific task against the time [25]. As our small study showed, a high-fidelity simulator can help to educate knowledgeable and confident specialists in a short period of time, at least regarding assesment of uterine cervix in pregnant woman. This strategy will lead to more accurate diagnostics and error-free health care in our specialty and allows standardized evaluation of training outcomes during the residency program.
